# Towards a New Dynamic Interaction Model of Adolescent CUD Manifestation, Prevention, and Treatment: A Narrative Review

**DOI:** 10.3390/psychoactives2040019

**Published:** 2023-10-17

**Authors:** Wesley Oosten, Elena Vos, Leontien Los, Michel Nelwan, Toine Pieters

**Affiliations:** 1 Freudenthal Institute, Utrecht University, P.O. Box 85 170, 3508 AD Utrecht, The Netherlands; 2 Trimbos Institute, P.O. Box 80 125, 3500 AS Utrecht, The Netherlands; 3 Department of Adolescent Psychiatry and Addiction Prevention, Brijder-Jeugd, 2553 NZ The Hague, The Netherlands; 4 Department of Children and Adolescent Psychiatry, Erasmus Medical Center Sophia, P.O. Box 2060, 3015 CN Rotterdam, The Netherlands; 5 Department of Pharmaceutical Sciences, Utrecht Institute for Pharmaceutical Sciences (UIPS), Utrecht University, P.O. Box 80 082, 3508 TB Utrecht, The Netherlands

**Keywords:** cannabis use disorder, cognitive development, adolescence, THC, drug, set, setting, individual and environmental risk factors, school performance, prevention, therapy

## Abstract

**Background:**

Cannabis is one of the most popular drugs of the 21st century, especially among adolescents and young adults. Evidence of a variety of lasting neuropsychological deficits as a result of chronic cannabis use has increased. Furthermore, regular cannabis use is found to be a predictor of mental health problems, less motivation in school, and school dropout.

**Aim:**

Our goal is to propose a theoretical model of adolescent cannabis use disorder (CUD) based on Zinberg’s drug, set, and setting model and explicated by a review of the literature on adolescent cannabis use to improve the prevention and treatment of CUD for adolescents.

**Methods:**

PubMed and Web of Science were searched for relevant publications as part of a hypothesis-based and model-generating review.

**Results:**

Individual (set) and environmental (setting) risk factors play important roles in the development of CUD in adolescents. School performance, motivation, and attendance can be negatively influenced by persistent cannabis use patterns and adolescent brain development can consequently be impaired. Thus, cannabis use can be understood as both being the cause of poor school performance but also the consequence of poor school performance. To prevent and reduce adolescent CUD the drug, set, and setting must all be considered. It is important to notice that the multiple feedback loops (indicated in our dynamic interaction model) are not mutually exclusive, but offer important intervention focus points for social workers, addiction professionals, parents, and other care takers.

**Conclusion:**

We argue that the three dimensions of drug, set, and setting contribute significantly to the eventual manifestation of CUD. Based on our dynamic interaction model, recommendations are made for possible preventive and therapeutic interventions for the treatment of adolescents and young adults with CUD.

## 1 Introduction

Cannabis is one of the most popular drugs of the 21st century, with an estimated 128−238 million users worldwide [1]. According to the Dutch Trimbos Institute, in 2022 one third of adolescents in the Netherlands have used cannabis at least once in their lifetime and about 1.5% of adolescent users can be classified as having a cannabis use disorder (CUD) [2]. Evidence has been found for both a variety of lasting neuropsychological deficits and decreased social well-being as a result of adolescent cannabis use [3–6]. In adolescence, a critical phase in life for brain development, individuals acquire new cognitive, physical, social, economic, and emotional resources. Importantly, adolescent well-being and health are essential for achieving future social well-being [7]. Regular cannabis use could be a threat to this important developmental period. Chronic adolescent cannabis use is associated with poor school performance and cognitive impairment [8]. The adolescent brain is particularly vulnerable to neurotoxic effects, especially regarding neurocognitive functioning. Furthermore, cannabis use is found to be a predictor of decreased school motivation, truancy, and school dropout [9]. It is widely recognized that there are potentially serious social-economic consequences for students who fail to complete their schooling [10].

Current treatment options for adolescents with CUD include cognitive behavioral therapy (CBT), multidimensional family therapy (MDFT), and motivational enhancement therapy (MET) [11]. Besides psychotherapies, pharmacotherapy trials are being conducted, with N-acetylcysteine being a potentially promising medication [12]. However, further research into N-acetylcysteine is needed as studies show mixed results [12,13]. Moreover, various studies have shown that a considerable proportion of adolescents with CUD do not benefit from current addiction treatment options [14,15]. Interventions are largely based on evidence from adult addiction treatment and research, yet adolescence is a developmental period in life characterized by unique social, neurobiological, and cognitive changes [16]. Therefore, to improve adolescent CUD treatment, clinical practice guidelines specifically for adolescent CUD should be developed.

We argue that to advance our understanding of adolescent cannabis use and the ways drug effects are co-shaped by social and cultural parameters, it is most fruitful to draw on the model of “drug, set, and setting” developed during the 1960s in the field of psychedelic drug research by the American psychologist Timothy Leary and psychiatrist Norman Zinberg. The concept of drug, set, and setting holds that the effects of drugs should be understood as the sum of these three basic elements [17]. “Drug” refers to the actual pharmacological action of the substance on the neurobiological system of a person. “Set” includes the personality structure (including psychopathology) of the user, the preparation, expectation, the intention, and the mood of the person at the time of use. The third factor that influences the effect of a drug is setting, which refers to the influence of the physical and social environment within which drug use occurs [18,19] While this concept emerged within the context of psychedelic research, it has also proven to be useful for researchers who have studied the effects of other medical and non-medical drugs [17]. For example, a recent study into opioid overdoses found that for thirteen out of twenty-nine participants, their overdose could be attributed to their set, and six to their setting. Thus, the authors posited that the set and setting must be considered when discussing drug use and overdose policies and interventions [20]. To our knowledge, Zinberg’s model has not yet been used in designing substance use disorder (SUD) treatments. Furthermore, the drug, set, and setting of adolescent cannabis use differ considerably from those of adults. Therefore, we use this model to explain specific risk factors that have an impact on the development of CUD during adolescence.

With this theoretical model proposal, we aim to contribute to the implementation of current knowledge in addiction treatment and emphasize the importance of clinical practice guidelines for adolescent cannabis use disorder (CUD) in which the drug, set, and setting are being considered. To begin, we will outline the development of the adolescent brain. In discussing adolescent brain development, we mainly focus on executive functions (EFs), as EFs and school performance are highly correlated [21]. Next, we discuss the model of drug, set, and setting and the role of these different dimensions in the manifestation of adolescent CUD. Third, we discuss the state of therapeutic intentions for adolescent CUD. Fourth, we propose a model based on drug, set, and setting in which cannabis use, EFs, and school performance are interrelated in a multidirectional way. Finally, recommendations are made for preventive and therapeutic interventions for the treatment of adolescents with CUD.

## 2 Materials and Methods

In identifying sources for this model generating review, the databases PubMed and Web of Science were used. The search terms consisted of: cannabis, adolescence, adolescent brain development, cannabis use disorder, CUD, CUD treatment, executive functions, school performance, cognitive development, tetrahydrocannabinol (THC), and individual/environmental risk factors. These terms were combined in various ways with “AND” commands. In addition, several articles were located through the reference lists of relevant articles (the snowball method). The inclusion criteria were: the scientific studies were from a peer-reviewed journal source; the year of publication was between 2015 and 2023; and the scientific articles were written in English. We did not use any specific exclusion criteria. As this is a narrative review, and not a systematic review, caution needed to be taken when interpreting the data. Select sources from the literature were added based on informal searches, so contradicting studies may have been missed. Despite being more than ten years old, Crone and Dahl’s model of adolescent brain development (2012) was chosen as a primary reference source for our study because it remains an exemplary model of adolescent brain development according to which affective and social influences interact with a broader set of changes in social cognitive development and cognitive control [22]. This is, in our view, the most suitable model for what we intend to investigate, but we also discuss more recent neurobiological findings on adolescent brain development.

## 3 Results

### 3.1 CUD and DSM

According to the DSM-5, a CUD diagnosis can be made when two of the eleven DSM-5 factors listed below have occurred in a 12-month period (see Table 1). The severity of the disorder is determined by the number of criteria fulfilled: mild: 2−3 symptoms; moderate: 4−5 symptoms; and severe: >5 symptoms [23]. However, it is important to realize that cannabis use can also be problematic even when an individual does not meet the DSM criteria for CUD. “Problematic cannabis use” may also involve (1) contra-indicating medical or psychiatric comorbidities (such as cardiovascular disease or a history of psychotic disorder) and (2) high-risk behaviors. Examples of non-CUD determinants for problematic cannabis use are poly-drug use, using high-potency cannabis, driving while intoxicated, and using cannabis ≥2 times/week during or before adolescence. It is therefore important to always consider the cannabis use parameters (frequency, potency, duration, etc.) on an individual level. Not meeting the CUD DSM criteria does not exclude cannabis use from being problematic for the individual [24].

### 3.2 Adverse Effects of Cannabis Use

Various adverse effects of cannabis use have been reported. A distinction can be made between the adverse effects observed in short-term use versus those observed in long-term or heavy cannabis use. Common adverse effects of short-term use (depending on the dose) include impaired short-term memory and motor coordination, altered judgement, hallucination, and paranoia. These effects make it difficult to learn and retain information and increase the risk of injuries by interfering with driving skills. Common adverse effects of long-term or heavy use include altered brain development, cognitive impairment, addiction, poor educational outcomes, symptoms of chronic bronchitis, increased risk of chronic psychosis disorders (in individuals with a predisposition for such disorders), and diminished life satisfaction and achievement [3]. Importantly, these effects are influenced by the age of CUD onset, the maximum daily use in terms of dosage, and the conditions of use [25]. Most of the effects of long-term/heavy use named above are strongly associated with cannabis use that begins early in adolescence. In addition, early and regular cannabis use is a predictor for increased risk of CUD. Individuals who begin to use cannabis in adolescence are approximately 2−4 times as likely to have symptoms of cannabis dependence within the first two years of use than those who begin use in adulthood. Likewise, the other impacts of altered brain development, cognitive impairment, poor educational outcomes, and diminished life satisfaction and achievement are all related to early initial use [3]. Another factor that plays an important role in the eventual effects of use is the potency of the cannabis. For example, the average THC level of Dutch home-grown cannabis (Nederwiet) is significantly higher than that of imported cannabis [26]. Compared with low-potency cannabis, high-potency cannabis appears to be associated with a greater risk of anxiety, depression, psychotic symptoms, and cannabis dependence [27]. In Section 3.4.1, effects of cannabis will be further discussed.

### 3.3 Adolescent Brain Development

Stages of development are marked by periods of significant brain and behavioral changes. Adolescence refers to the stage of development between childhood and adulthood between the ages of ten and nineteen and is characterized by unique social, neurobiological, and cognitive developments [28]. Higher-order cognitive abilities improve and corresponding refinements of the function and structure of the brain regions that support them take place [16]. There is increased connectivity between brain regions, changes in white and grey matter, and increased dopaminergic activity in the limbic system, the striatum, the prefrontal cortices, and the pathways linking them [29].

Importantly, the frontal cortical areas of the brain, which control higher-order cognitive processes such as response inhibition and working memory, are the last to reach full maturity. These higher-order cognitive abilities are also referred to as executive functions (EFs) [30]. EFs can be conceptualized as being essential in the self-regulation of behavior [31]. Self-regulation, as Barkley states, involves any action an individual directs at oneself to change behavior to attain a goal or specific consequence. Executive functioning includes multiple cognitive processes such as problem-solving, inhibitory control, cognitive flexibility, and planning [1]. Furthermore, a robust correlation between EFs and academic performance has been found. EFs assessed in early childhood have been found to predict grades, readiness for both math and reading, high school completion, overall school achievement, and college graduation. EFs even predicted outcome better than IQ in many cases [21]. A comprehensive study among 2036 individuals aged five to seventeen was performed to examine age-related changes in EFs and their relation with academic achievement. In this study, three EF tasks, nine academic tests, and several aspects of performance (ratio, accuracy, and completion time) on the EF tasks were included. The correlation between academic achievement and EFs varied across ages, but there was a remarkable similarity in the strength of these correlations for overall reading and math achievement as part of the developmental pattern. This result suggests a domain-general relation between academic achievement and EFs [32].

**Figure 1:**
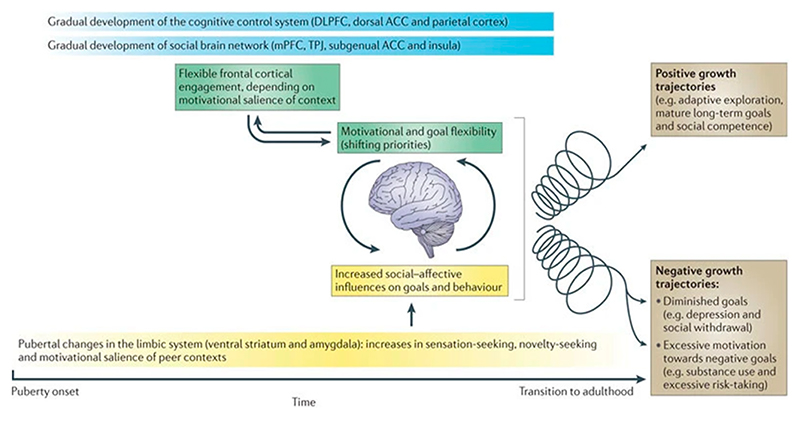
The model of adolescent brain development proposed by Crone and Dahl (2012). Reprinted from “Understanding adolescence as a period of social−affective engagement and goal flexibility” by Crone and Dahl [22].

Crone and Dahl (2012) proposed a model of adolescent brain development according to which affective and social influences interact with a broader set of changes in social cognitive development and cognitive control (see Figure 1) [22]. These changes include the acquisition of cognitive (executive function) and social control skills that develop gradually over adolescence. The interaction between cognitive control systems and social−affective processing systems contributes to flexibility in the engagement of frontal cortical systems in adolescents, depending on the motivational salience of a goal. Also, dynamic changes in the mesolimbic dopamine pathway, which regulates reward-associated processes, take place during adolescence [33]. Because of these changes, adolescents have an increased tendency to take risks, try new things, and explore and have increased social motivation. Although these behavioral tendencies may be seen as impulsive, the capacity to quickly shift goal priorities may also enable individuals to effectively engage cognitive systems in situations when highly motivated to do so. Importantly, Crone and Dahl predict that this increase in social−affective engagement not only influences behavior and incentives in the moment but also the patterns of behavior and motivation learning over longer intervals [22]. Quickly shifting priorities according to social incentives can, over time, contribute to healthy exploration and risk-taking behaviors. This behavioral tendency can promote emotional and social learning and the development of knowledge and skills that underpin adult social competence. However, when motivational learning processes and risk-taking come in response to unhealthy incentives, such as thrill-seeking or drug abuse, these same tendencies can have negative consequences. Thus, according to this model, flexible prefrontal cortex engagement in combination with changes in social−affective processing is generally adaptive but can also contribute to negative consequences through the interaction between individuals risks and certain environmental factors [22].

In line with Crone and Dahl’s model, the life-span wisdom model (LSWM) posits that risk taking during adolescence is adaptive because being exposed to novel experiences furthers the development of wisdom [34]. However, according to the LSWM, some adolescents who have high sensation-seeking tendencies also exhibit higher impulsive behaviour tendencies, which may predispose them to continue using drugs and increase their risk of substance use disorders (SUDs) [35]. Furthermore, recent neurobiological findings show that during adolescence, there are neural changes in brain areas involved in affective processing, cognitive control, and social cognition that influence the relationship between adolescents’ social environment and risk taking [36]. In short, individuals experience a heightened sensitivity to rewards due to a developmental peak in reward-related brain areas, while the prefrontal cortex, involved in self-regulation, continues to develop into early adulthood [37,38]. The mismatch between reward sensitivity and self-regulation is suggested to be an important factor in risk taking in adolescence, and thus in the risk of developing a SUD [39]. Additionally, a recent study by Goddings et al. (2023) used functional magnetic resonance imaging (fMRI) to investigate the relationship between puberty and neural activation during risky decision making. The results suggest a puberty-related shift in neural activation within brain areas involved in processing the outcomes of risky decisions that might reduce adolescents’ sensitivity to negative feedback and subsequently contribute to increases in risk-taking behaviors [40].

In summary, the brain continues to develop during adolescence, and while rewardrelated and affective brain areas reach their peak, the frontal cortical areas involved in self-regulation are not fully developed until early adulthood. These neurobiological changes can, in combination with social influences, lead to both adaptive goal-shifting and healthy exploration and risk taking (e.g., using substances).

### 3.4 Drug, Set, and Setting of Cannabis Use

#### 3.4.1 Cannabis (Drug)

##### Mechanism of Action

While consisting of more than 400 different active constituents, the cannabinoid Δ9-tetrahydrocannabinol (THC) is the primary psychoactive constituent of the Cannabis sativa plant. THC is believed to be primarily responsible for the addictive potential and cognitive effects of cannabis [41]. Another major cannabinoid in cannabis is cannabidiol (CBD), which is a potent anti-inflammatory agent that causes a variety of other effects [33,42].

The neurobiological mechanism underlying the effects of cannabis is the endogenous cannabinoid system or endocannabinoid system (ECS). The ECS has a regulatory role in neurotransmitter release. The two main receptors of this system are the cannabinoid CB1 and cannabinoid CB2 receptors. The CB1 receptor is a pre-synaptic heteroreceptor that can primarily be found in the central nervous system and is abundant in brain areas associated with emotional responses, motivation, and motor control. The CB2 receptor is primarily located in the peripheral and immune tissues. Both receptors may be activated by either endocannabinoids (naturally generated inside the body) or cannabis and cannabis-related synthetic compounds [42]. The neuronal activity of other transmitter systems, such as the glutamatergic, dopaminergic, and GABAergic system, can be modulated by the ECS. The ECS is a retrograde messenger system that regulates both inhibitory and excitatory neurotransmission according to an on-demand principle [33]. It has been implicated in a wide variety of behavioral functions such as various cognitive functions, the regulation of fear and anxiety-related behavior, and the modulation of the effects of drugs of abuse [30,43]. Moreover, the ECS is considered to play an important role in higher-order brain functions, including executive function, which continue to develop during adolescence [30,44].

THC exhibits partial agonistic activity for both CB1 and CB2 receptors [45]. It has been shown that THC administration can disrupt the endocannabinoid-mediated regulation of synaptic transmission [1]. CBD has low affinity for both CB1 and CB2 receptors and is a negative allosteric modulator that reduces the binding of CB1 agonists. CBD has been shown to have beneficial neuroprotective, anxiolytic, antipsychotic, and anti-inflammatory properties, though the underlying mechanisms for these effects remain elusive [46]. Many of the psychological effects of cannabis are biphasic, thus depending on the dose level, the THC/CBD ratio, and the user. Due to these biphasic effects, cannabis can either lead to relaxation and euphoria or anxiety and dysphoria. Anxiety due to cannabis use appears to be related to the THC level of the dose as well as the anxiolytic action of CBD. A high dose of CBD combined with THC reduces the intoxicating effects of THC [47].

Importantly, the ECS reaches peak activity and expression during adolescence and is involved in fine-tuning the mesolimbic dopamine pathway, which is involved in regulating reward-associated processes [33]. This makes the ECS a key modulator of adolescent developmental processes involving the mesolimbic reward circuitry and, subsequently, a key modulator in vulnerability to drug addiction. THC leads to elevated dopamine levels in the mesolimbic dopamine system [33]. Therefore, it is possible that the use of cannabis during adolescence can disrupt normal brain development and increase vulnerability to drug addiction [48].

##### Effects of Cannabis Use

Various effects of cannabis have been reported, some of which are positive and some negative. Here, we first outline some general positive and negative effects of cannabis use and then turn the focus to adolescence. Research shows that cannabis may have positive effects on symptoms associated with neurological disorders, such as multiple sclerosis (MS) and chronic pain [49]. For example, cannabis-based medication can improve subjective alleviation of MS symptoms and improve appetite and sleep [49,50]. Moreover, research among 274 participants with an average age of 51.2 years with treatment-resistant chronic pain showed that medicinal cannabis improved pain symptom scores, pain severity scores, and pain interference scores. In addition, social and emotional disability scores improved and opioid consumption decreased [51]. Cannabis is also used to treat sleep disorders. However, whereas cannabis improves sleep in patients with pain-related disorders, there is no benefit for healthy participants’ sleep. While participants report subjective improvements in sleep, there are no objective improvements found in sleep patterns [52]. In short, evidence shows that cannabis might be of therapeutic value. However, the studies all focused on adults as we could not find any research on the therapeutic value of cannabis specifically for adolescents. Also, further research into the most effective type of cannabis, the dose, and the mode of administration for different therapeutic indications is recommended.

Besides promising therapeutic effects, adverse effects of cannabis use have been reported. A distinction can be made between adverse effects observed in short-term use versus those observed in long-term or heavy cannabis use. The adverse effects of shortterm use include impaired short-term memory and motor coordination [53], altered judgement [54], hallucinations, and paranoia [3,55]. These effects can interfere with learning and retaining information and increase the risk of injuries by interfering with driving abilities [54,56]. Adverse effects of long-term or heavy use include altered brain development, cognitive impairment, poor educational outcomes, increased risk of psychosis [57], and diminished life satisfaction and achievement [3]. Individuals who use cannabis have a one in five risk of developing CUD [58]. Moreover, Blanco et al. (2016) showed that individuals who used cannabis in the last twelve months had a higher chance of developing any mood, anxiety, and/or SU disorder than individuals who did not use cannabis [59].

Importantly, the adverse effects of cannabis are influenced by the age of cannabis use onset, the maximum daily use in terms of dosage, and the conditions of use [25,57]. Most of the effects of long-term/heavy use named above are strongly associated with cannabis use that begins early in adolescence. In addition, early and regular cannabis use is a predictor for increased risk of CUD. Individuals who begin to use cannabis in adolescence are approximately 2−4 times more likely to have symptoms of cannabis dependence within the first two years of use than those who begin use in adulthood [60]. Likewise, altered brain development, cognitive impairment, poor educational outcomes, and diminished life satisfaction and achievement are all related to early initial use [3]. Another factor that plays an important role in the eventual effect of using is the potency of cannabis. For example, the average THC level of Dutch home-grown cannabis (Nederwiet) is significantly higher than that of imported cannabis [26]. Compared with low-potency cannabis, high-potency cannabis appears to be associated with a greater risk of anxiety, depression, psychotic symptoms, and cannabis dependence [27].

In short, besides therapeutic effects, cannabis can have serious adverse effects. Adolescents and young adults need to take special caution when using high (THC)-potency cannabis products and be aware of the additional risks of starting cannabis use at an early age of onset on a regular basis and with a family history of mental health problems.

##### Neuropsychological Effects of Cannabis Use

As the brain continues to develop throughout adolescence, cannabis may influence neuropsychological development and functioning. In view of the increased sensitivity of the cannabinoid system and the ongoing maturation of particularly the frontal regions of the brain during adolescence, exogenous cannabinoids could disrupt normal brain development and have an impact on cognitive function [8]. Indeed, several animal and human studies have provided evidence for the association between cannabis exposure during adolescence and connectivity and morphological changes in brain structures that are densely populated with cannabinoid receptors (e.g., in the hippocampus, cerebellum and prefrontal cortex (PFC)) [61,62]. Weiland et al. (2015), however, found no association between daily cannabis use and morphological changes in brain structures in adolescents [63]. Although research into the relationship between cannabis use and morphological/structural changes is inconclusive, various studies have shown that long-term and heavy cannabis use is associated with impaired neurocognitive functioning in animals and humans [64–66]. For example, studies comparing adolescents using cannabis on a regular basis with a control group reported that they performed more poorly on tasks assessing verbal memory [67,68], intelligence [69], attention [67,68], and executive functions [68–70] and that they have a reduced processing speed [68,70]. However, these results should be treated with caution as it is not entirely clear how and to what extent cannabis affects mental functions. For example, THC seems to interfere with the encoding of verbal memory without interfering with retrieval, suggesting that cannabis does not influence learned information prior to the use [71]. Moreover, there is no consensus about the way cannabis use and impaired executive functions (EFs) are related. One mechanism by which poorly developed EFs may increase the risk of cannabis use is through more impulsive risk-taking and externalizing behavior, which is associated with substance use. Gustavson et al. (2017) found that impaired EFs were indeed a risk factor for early aspects of (poly)substance use in adolescence. However, non-EF factors, such as genetic factors that influence the subjective effects of substances, play a larger role in the actual progression to substance dependence [72].

Whether or not the neuropsychological effects of cannabis persist after extended periods of abstinence is still debated. Some studies have reported (subtle) lasting neuropsychological deficits in adolescent cannabis users compared to non-users [68,73]. For example, overactive brain regions involved in higher-order cognitive processes [74] and the default mode network—a set of regions in the brain that are active during passive tasks and possibly involved in the capacity to imagine future actions or events [75]—were seen in cannabis users compared to a control group after a period of abstinence [74]. These results indicate a vulnerability of the adolescent brain to residual effects of long-term cannabis use [48]. However, meta-analyses have shown that no residual, non-acute effects of cannabis use on cognitive performance in adolescents and adults were detectable after one month or more of abstinence [76,77]. Factors that affect the impact of cannabis use are the magnitude and frequency of use, medical versus recreational use, and the length of abstinence [78].

An important limitation is that most studies contributing to this debate are retrospective or cross-sectional case−control, without proper assessment of cognitive function prior to the onset of cannabis use. To clarify temporal associations between neurocognitive development and cannabis use and guide prevention efforts, prospective longitudinal studies are needed that include pre- and post-drug use neurocognitive assessments [8].

#### 3.4.2 Individual Motives and Risk Factors (Set)

In the 2022 American “Monitoring the Future” survey of high-school students, 3.2% of 13−18 years old students reported daily cannabis use, and 31% of 12th grade students reported cannabis use in the past 12 months [79]. The 3.2% might even be an underestimation of daily use, since students who have dropped out of school may have particularly high rates of daily cannabis use. Interestingly, an American study showed that about 80% of 12,024 12- to 17-year-old adolescents reported perceived risk of harm from monthly cannabis use [80]. To understand why adolescents use cannabis, individual motives and risk factors for adolescent cannabis use, described as the set in the drug, set, and setting model, will be discussed.

The literature shows that the five most common and most researched motives for adolescent cannabis use are coping, conformity, sociocultural engagement, self-medication, and expansion [81–83]. Defoe et al. (2022) reviewed adolescents’ own views on their risk behaviors, including cannabis use, and they found that the most frequently mentioned motives by adolescents were being cool/tough, enjoyment, belonging, having fun and experimenting, and coping [84]. Coping as a motive for cannabis use is particularly related to more problematic use and greater anxiety and depressive symptoms [82]. Adolescence can be a stressful transition period from childhood to adulthood. New social and intellectual skills must be learned, which can lead to a great deal of stress. Adolescents having difficulties in coping with stress are at higher risk for using cannabis to cope [85] and developing a SUD [86]. However, the relationship between stress and cannabis use might be more complex as research shows that depression and anxiety mediate the relationship between perceived stress and problematic cannabis use [87].

Further, research shows that poor performance on executive function and IQ tasks early in life is associated with substance use risks later in life and increased cannabis use frequency [88,89]. However, this association may be mediated by other factors, such as sociocultural factors or personality traits. One personality trait that is consequently found to be associated with adolescent and young adults’ cannabis use is neuroticism [90,91]. Chowdhury et al. (2016) suggested that high levels of neuroticism can result in heightened sensitivity to arousal, which may in turn increase the motivation to use cannabis to alleviate arousal [92]. Moreover, Dash et al. (2019) showed that neuroticism was positively associated with CUD in 3.785 twins and siblings [93]. Note that the age range in this study was 21 to 46, so the results are not directly generalizable to adolescents. The results, however, do suggest an interesting finding, namely that neuroticism is associated with CUD somewhat independently of shared genetics and environmental factors.

Moreover, compromised mental health and substance abuse disorders often coexist [86]. For example, a strong association is shown by the scientific literature between posttraumatic stress disorder (PTSD) and SUD. In both male and female substance-using populations, high rates of trauma exposure have been observed. Up to 60% of the individuals in clinical trials who sought treatment for SUD even met the diagnostic criteria for PTSD [94]. Furthermore, studies found that individuals with elevated social anxiety have increased risk for SUD and greater cannabis-related problem severity. Social anxiety is comprised of sub-facets, e.g., social avoidance. Individuals with social anxiety may use substances to attempt to cope with one or more of these facets despite substance-related problems [95]. In addition, several neurodevelopmental disorders are a predisposition for cannabis use or dependence [96,97], such as attention-deficit/hyperactivity disorder (ADHD) and autism. Cross-sectional and longitudinal studies show a significant increase in the risk for cannabis use, abuse, and dependence for adolescents with ADHD and autism [97]. In short, mental health is important to consider when discussing any substance use disorder, including CUD [98].

Lastly, genetics play an important role in the development of SUDs. Although heritability estimates vary across studies and types of SUDs, twin and family studies have shown that genetics account for roughly 50% of the risk for developing a SUD [99]. Specifically for CUD, twin studies show that heritability estimates range from 0.51 to 0.59, meaning that genetics account for 51−59% of the risk for developing CUD [100]. However, genes only partly explain the eventual manifestation of SUD and CUD [101,102]. Environmental factors seem to largely determine whether and when a person starts to use a substance, after which genetic factors become important in predicting how much a person will use and to what extent they will show problematic addictive behavior [103]. Further, gene expression is suggested to differ by stage of SUD, such as substance initiation versus chronic use [99]. Other known individual risk factors for SUD are parental history of SUD, early onset of smoking, and disruptive behavior in childhood [104].

#### 3.4.3 Environmental Risk Factors (Setting)

According to the drug, set, and setting model, the eventual effect of drug use is the result of an interaction between the drug itself and individual and environmental factors. Environmental factors (setting) can be physical and social [18]. Well-known social environmental factors that can contribute to risk behaviors include peer groups and family. When adolescents are surrounded by negative influences, such as deviant peers and weak bonds, these adolescents face challenges. Adolescents who are resilient can overcome such challenges; however, for some individuals these stressors may lead to substance use and abuse [86,105,106]. Conversely, peers and family can also be important protective factors [107]. Moreover, a supportive school environment is essential in both promoting learning and combating anti-social behavior, such as substance use, among adolescents. According to the stage-environment fit theory, students experience the highest levels of well-being and are most motivated when a school social climate meets their socio-emotional needs [108]. Receiving attention and empathy generate a sense of belonging that leads to increased academic motivation and engagement. Specifically, the most significant aspect of the school social climate seems to be students’ perception of their relationship with others (students and teachers) at school [108]. Conversely, poor school social climates are found to be a strong negative predictor of the frequency of cannabis use. For example, pressure from teachers and parents to excel academically can make the academic environment stressful [86]. In addition, there is an association between peer substance use and an adolescents’ own substance use. Longitudinal research shows that having peers who use substances can lead to increased substance use and one’s own substance use can lead to selecting peers who also use substances [109]. For example, adolescents with ADHD and anxiety symptoms and autistic traits may have difficulty connecting with neurotypical classmates and tend to opt out in favor of like-minded cannabis-smoking and often mellow peer groups [97].

Furthermore, lower academic achievement is found to be positively associated with maximum daily use, younger age of cannabis use onset, and regular cannabis use [25]. Cross-sectional studies have shown an association between increasing levels of cannabis use and poor school performance, negative attitudes towards school, less satisfaction with school, and increased school absence [110]. However, cross-sectional studies cannot determine causality, so longitudinal or experimental research is needed to examine the direction of the relationship between cannabis use and educational performance. One longitudinal study by Meier et al. (2015) showed that adolescents who persistently used cannabis had lower GPA scores in twelfth grade than was predicted, even when controlling for ninth-grade GPA [111]. This suggests that persistent cannabis use may cause decreased educational performance. Schuster et al. (2018) sought to determine whether cognition improves because of cannabis abstinence [112]. Adolescents and young adults who regularly used cannabis were randomized to an abstinence condition or a monitoring control condition to control for group differences that might influence performance (e.g., learning ability). The results showed that one week of cannabis abstinence was associated with improvements in verbal learning, suggesting that cannabis use temporarily causes poorer educational performance. However, this is just one study and there was no effect of abstinence on attention found [112]. All in all, the question remains whether poor educational performance is a consequence of cannabis use or vice versa.

Another socio-cultural environmental factor is the availability of cannabis. In the Netherlands, coffee shops can sell cannabis under strict conditions, and individuals can carry up to 5 g of cannabis with them without being prosecuted [113]. The tolerance policy and mass supply of cannabis may be associated with higher consumption rates and a younger age of initial use [114]. However, while the Netherlands was one of the European countries with the highest prevalence of adolescent cannabis use in 2019, its incidence of CUD was among the lowest in Europe [115]. This suggests that the relationship between cannabis use and problematic cannabis use is influenced by broader cultural and social-economic factors.

Finally, the global COVID-19 pandemic was an environmental factor with enormous impact. Measures taken to contain the spread of the virus (e.g., closing schools and curfews) required flexibility. The sudden transition to emergency online education in early 2020 was perceived by teachers, students, and parents alike to be a severe disruption. Mental stress among others was observed to rise sharply [116]. Distance learning was a novel situation for students, who now had to self-regulate and organize their learning autonomously. The research of Pelikan et al. (2021) pointed out the importance of physical education; they found that a subgroup of students who perceived themselves as low in digital competency were less able to cope with the situation of distance learning [117]. Gaps in access to education between different social-economic groups also widened. Given that lower academic achievement, motivation, and well-being of students are associated with cannabis use, these results further demonstrate the importance of a (supportive) school environment, especially during a pandemic that already seems to lead to a higher risk for substance abuse [116–118].

In short, environmental factors may influence adolescent cannabis use and are therefore important to consider in designing prevention and intervention programs for adolescent CUD.

### 3.5 Landscape of Therapeutic Interventions

In the Netherlands, roughly 16% of the 55.000 individuals who experience problems because of their cannabis use are in treatment. Most individuals are male, and the mean age is 32 [119]. To our knowledge, there is no information available about the percentage of adolescent cannabis users who seek treatment. Dennis et al. (2014) estimated that 90% of adolescent heavy users never receive treatment [120]. This may be explained by individuals’ perceptions of risk, low motivation to change, and stigma. Adolescents who do engage in treatment are often referred to addiction centers by their parents, school administration, or general practitioner. Many of these individuals, however, have little intrinsic motivation to change their behavior, which hinders effective treatment [121].

A distinction can be made between therapeutic interventions for adolescents who engage in treatment and interventions for non-treatment seeking adolescent cannabis users. Treatment options for treatment-seeking adolescents with CUD include cognitive behavioral therapy (CBT), multidimensional family therapy (MDFT), and motivational enhancement therapy (MET) [11]. A 2011 Dutch RCT randomly assigned 109 adolescents from substance abuse treatment sites to either MDFT or CBT treatment of five to six months [122]. CBT focuses on the conjunction of thoughts, feelings, and behavior and is based on the principles that changing thinking patterns and/or behavioral patterns lead to more adaptive and less maladaptive behavior. This study tried to enhance the patients’ motivation to change their addictive behavior through self-control training, social and coping skills training, and relapse prevention. MDFT is a family-based treatment for adolescent SUDs that focuses on improving four major domains, namely the adolescent themself, their relationship with their parents, their relationship with other family members, and their relationship with the community (e.g., peers and school). The results show that adolescents in both treatment groups showed significant reductions in cannabis use and that CBT and MDFT were both equally (moderately) effective [122]. MET is based on techniques used for motivational interviewing (MI) and involves enhancing motivation to change behavior by means of providing nonjudgmental feedback, resolving ambivalence, and collaborative goal setting [11]. MET has been shown to be effective for treating adolescents with SUDs [123]. This is in line with a meta-analysis showing that MI was efficacious in achieving abstinence in adolescent cannabis users [124].

Interventions for non-treatment seeking adolescent cannabis users are often brief and school-based interventions that focus on motivational interviewing techniques. A 2022 systematic review revealed that most brief interventions had a mild positive effect on cannabis consequences but did not lead to a significant reduction in cannabis use [125]. One RCT among 252 non-treatment-seeking adolescents looked at whether a change in motives was associated with reductions in cannabis use. Participants received two sessions of MET and had three check-ins. The results show that the intervention led to significant reductions in motives for use and that this change was associated with reductions in use and problematic outcomes [126]. This suggests that a change in motives may be necessary to achieve successful intervention effects.

In short, several treatment options exist for adolescents with CUD. However, various studies have shown that a considerable proportion of adolescents with CUD do not benefit from current treatment options [14,15]. Finally, all treatment options mainly focus on the set, with little attention being paid to the drug and the setting. Therefore, there is a need for the development of effective treatments that consider the drug, set, and setting dimensions of CUD.

### 3.6 Integrated Dynamic Interaction Model

The literature reveals that cognitive functioning and school performance are important psychosocial determinants for adolescent cannabis use. The relationship between cannabis use in adolescence, cognitive functioning, and school performance is multifaceted and multidirectional. School performance, engagement, absence, and motivation in this context are being simplified under the umbrella of “school performance”. Thus, school performance is not only about students’ grades, but also their motivation to participate as well as their presence and peer group interaction at school. Here, we discuss the interaction between cannabis use, EFs and school performance, after which we propose a theoretical model for adolescent cannabis use.

#### 3.6.1 Interaction between Cannabis, EFs and School Performance

Several prospective longitudinal studies have shown a significantly increased risk of poor school performance and leaving school early because of early cannabis use [112,127]. One possible explanation for this is that cannabis use encourages anti-conventional behavior and retreat into like-minded peer groups of dropouts, which could lead to failure to learn at school [110]. This may interfere with the capacity to achieve the increasingly challenging educational goals that are expected from students at school. Another explanation is that possible long-lasting cognitive impairments, due to long-term and heavy cannabis use, lead to poor educational performance. As discussed above, cannabis use during adolescence is suggested to result in long-lasting and measurable cognitive impairments, particularly if use begins during early adolescence [48,68,73].

Following a neuro-constructivist approach [127], we propose that cannabis use interferes with normal brain development in a critical period, during which executive functions unfold. This interference might disrupt the pathway to obtain the necessary self-regulatory capacities used for academic performance, causing individuals to fall into a deficit with growing environmental demands. Cognitive deficits caused by cannabis use are, therefore, expected to be related to lower academic achievements [3]. However, as previously stated, cannabis use and poor EFs may also be related to shared genetic influences. Likewise, poor EFs could be a risk factor for cannabis use [72]. In summary, an association between cannabis use and poor school performance has been shown, which is hypothesized to be either directly caused by cannabis or mediated by cognitive impairment as a result of cannabis use [8].

However, the opposite could also be true: poor school engagement could possibly lead to more cannabis use [128]. Because adolescent cannabis users are less likely to complete school, their cognitive development may also be affected [129]. Longitudinal studies have provided evidence for this reverse causal association [8,130,131], but not all studies confirm this evidence for reverse causal pathways in which lower educational achievement leads to increased cannabis use, after adjusting for pre-existing levels of cannabis use and background factors [132].

Thus, it is likely that poor educational performance can be a consequence of cannabis use, and that poor educational performance can lead to cannabis use, or that both could materialize and influence each other simultaneously. It is also possible that the relation between cannabis use, and educational performance is partly, or even entirely mediated by other factors, such as socio-emotional or environmental elements [133]. Jackson et al. (2016) examined the relationship between cannabis use and changes in intellectual performance within two longitudinal studies of adolescent twins (*n* = 789 and *n* = 2277). Cannabis users had lower test scores than non-users, however, cannabis-using twins showed no significant IQ decline relative to their non-using sibling. These results suggest that the decline in measured IQ was not directly contributable to cannabis use, but rather to familial factors that underlie both cannabis use and low intelligence [134].

#### 3.6.2 Model of Interaction

**Figure 2:**
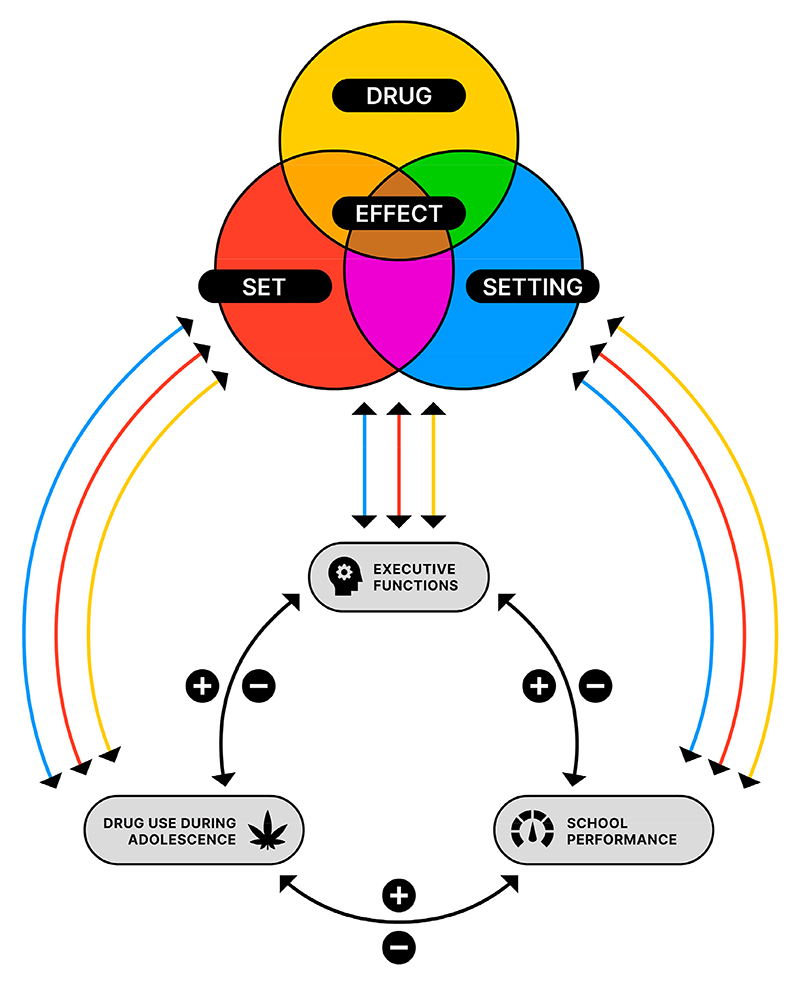
Interaction between drug use during adolescence, executive functions (EFs) as part of adolescent brain development, and school performance. Within this model, the positive (+) and negative (−) correlations between the factors are visualized. Also, the drug, set (individual factors), and setting (environmental factors) of an individual independently influence the drug use, executive functions, and school performance.

In Figure 2, we propose a dynamic interaction model in which cannabis use, adolescent brain development, and school performance are interrelated in a multidirectional way. Within this model, the positive (+) or negative (−) correlations between the factors are visualized. Moreover, drug, set, and setting are visualized as three different dimensions that can independently contribute to use of cannabis, executive functions, and school performance. For example, more and sustained cannabis use can cause impaired EFs and poor educational performance. It is important, however, to realize that this also works the other way around: less cannabis use can have a positive influence on the other factors.

To summarize, early cannabis use can be associated with poor school performance and motivation and increased absence rates. This could be explained as cannabis use being the cause of poor school performance (with the mediation of poor EFs) but also as cannabis use being the consequence of poor school performance. It is important to notice that these feedback loops are not mutually exclusive [8,128], and that the interrelationship between the various risk factors may be more complex [133,134]. Heavy and long-term cannabis use has been linked to anti-social behavior, lower satisfaction with life, lower income, and greater need for socio economic assistance [132,135]. All these factors could possibly influence school performance. Moreover, the drug, set, and setting contribute significantly to these three factors independently and to the eventual manifestation of CUD.

## 4 Discussion

The multidirectional relation between adolescent cannabis use, adolescent brain development and school performance is influenced by the properties and mechanisms of cannabis and individual and environmental risk factors. During adolescence, the combination of changes in social−affective processing and flexibility in PFC recruitment is generally adaptive and developmentally appropriate to the learning demands and tasks of adolescence, but it can also create vulnerabilities to engage in negative behaviors in some incentive situations [24]. In view of the increased sensitivity of the cannabinoid system and the ongoing maturation of particularly frontal regions of the brain during adolescence, exogenous cannabinoids could disrupt normal brain development and have an impact on cognitive function [8]. The eventual effect of cannabis use depends on the THC/CBD ratio, dosage, and potency of the cannabis [47]. Furthermore, the set is important to consider since mental health and the age of onset of cannabis use are important risk factors for developing CUD [59,60]. Other examples of intra-individual factors that are a predisposition for long-term early onset cannabis use are genetics [100], neuroticism [93], the inability of coping with stress and anxiety [85], experiencing trauma, mental health problems [59], and neurodevelopmental disorders such as ADHD and autism [97]. Environmental factors that have an impact on the severity of adverse effects of cannabis use are relationships with peers and parents [86], availability of cannabis [26,114], the school environment, and the support of teachers [86]. Current preventative and therapeutic intervention options are CBT, MDTF, and MET. However, a considerable proportion of adolescents with CUD do not benefit from current treatment options [14,15]. Therefore, there is a need for the development of effective treatments that take into account the cognitive and social development of adolescents, and consider the drug, set, and setting dimensions of CUD.

### 4.1 Prevention-Based Interventions

It has been estimated that rates of adult substance abuse and dependence could be reduced by up to 10% with every year that onset of regular drug use is delayed in adolescence [136]. Hence, early-age prevention-based interventions are important. It is important to realize that even small effects of adolescent intervention efforts could result in significant health benefits on a population level [137].

When designing prevention and intervention programs, policy makers should consider the importance of integrating the different dimensions of drug, set, and setting. We propose an integrated approach with a simultaneous focus on the drug itself, the individual risk profile, and the environmental risk factors. Individuals should better be informed about the impact of these different dimensions on the development of substance dependence. Regarding the drug itself, it is important to mention that the levels of THC and CBD in cannabis can differ significantly between samples and that different THC/CBD ratios can cause different effects [47]. Conrod et al. (2010) investigated the efficacy of targeted coping skills intervention on drug use in adolescents. The cannabis intervention appeared to be associated with a nonsignificant trend for reduced odds of cannabis use. Calculations indicated that for every eighteen adolescents who participated, one case of cannabis use was prevented. The authors suggested that the intervention was not very successful due to the increasing trend that adolescents view cannabis use as favorable and less harmful than other drugs [137]. One study among 12.024 adolescents showed that individuals who did perceive monthly cannabis use as being risky had high parental monitoring, low perception of peer use, high perception of peers’ disapproval of cannabis use, high perception of school importance, and engaged more in extracurricular activities [80]. Therefore, cannabis education in schools that addresses problematic drug attitudes and informs adolescents about the adverse effects cannabis might have, especially considering the adolescent brain, is recommended. Also, by explaining that the brain undergoes neurobiological changes during adolescence, and how these changes, in combination with social influences, promote healthy exploration but can also increase risk taking, might help adolescents to become more aware of their decision making. Furthermore, current intervention programs seem to focus little on the combination of individual and environmental risk factors [138]. An overview of studies on preventing adolescent cannabis use revealed that effective interventions are school-based and integrated into classes or are peer-led including group motivational approaches [139]. Family interventions and motivational interviewing in non-school settings also seem to be effective [140].

Thus, we propose school-based prevention interventions that are integrated into classes and are semi peer-led. During these interventions, attention must be paid to the combination of individual and environmental risk factors, and more knowledge about the adolescent brain and cannabis risk awareness among care takers and adolescents must be created [141]. This approach is substantiated by research showing that adolescents who perceive that cannabis use is risky have high parental monitoring and low perception of peer use [80].

### 4.2 Therapeutic Interventions

Based on our review study, several observations and recommendations can be made about therapeutic interventions used for treatment of adolescents with CUD. First, there is an urgent need for clinical practice guidelines specifically for adolescent CUD or SUD in general. Current interventions for adolescents are mainly focused on the set and mostly based on addiction treatment for adults. However, adolescence is a unique period with social, neurobiological, and cognitive developments and this means that it is worth investing in the development of effective treatments that consider the drug, set, and setting dimensions of CUD (regardless whether the effects of cannabis are reversible) [68,73].

We would like to emphasize the importance of an integral approach. Interventions that are customized based on the individual’s circumstances are particularly needed. Most Dutch adolescents with CUD are referred to addiction centers in the Netherlands by parents, school administrations, and general practitioners and have little self-motivation to stop cannabis use [142]. For adolescents with CUD, their cannabis-using peer group often represents a social refuge culture with like-minded and supportive peers. The first treatment challenge is therefore to connect with the specific set and setting of the adolescent who uses cannabis to awaken the inner drive to act. Motivational enhancement therapy (MET) as a first-choice treatment modality can help individuals resolve their ambivalence about engaging in treatment and start regulating their drug use [121]. Further, studies show that a motive for adolescent cannabis use is coping, and that coping as a motive is particularly related to more problematic use and mental health problems [82]. Acceptance and commitment therapy (ACT) is a psychotherapy that helps individuals accept their thoughts and feelings and deal with them more flexibly [143]. We suggest that by targeting maladaptive coping and mental health through ACT, cannabis use will subsequently decrease. A review of the use of ACT in treating SUDs, including CUD, confirms that ACT is effective in reducing substance use and, in some cases, the discontinuation of use and subsequent abstinence [144]. MET and ACT can be used individually or combined, possibly in addition to a gradual THC-dosage reduction and self-monitoring of physical and mental conditions. Subsequently the set and setting could be addressed by providing multidimensional family therapy and stress coping strategies as part of cognitive behavioral therapy [145]. Thus, by paying attention to the specific combination of drug, set, and setting of an individual while also considering the most important psychosocial determinants in the cognitive and social development of adolescents, effective treatment of CUD is most likely to be achieved. However, further studies of this kinds of tailor-made therapeutic interventions are necessary and should include long-term follow-up research protocols to investigate the effectiveness of the data over time.

### 4.3 Limitations and Future Research Recommendations

This review paper gives an overview on adolescent cannabis use disorder. The major strength of this paper is that we viewed adolescent CUD through the lens of Zinberg’s drug, set, and setting model and thereby emphasizing specific risk factors that have an impact on the development of CUD during adolescence. However, this paper has several limitations which need to be noted. First, this is a narrative review and not a systematic review. We did not have a predefined search strategy and the method lacked strict inclusion and exclusion criteria, which may have caused selection bias. Second, we used English-written papers but we did not select by country, which may influence the results as cannabis law and legislation differ per country. Finally, this paper focused on adolescent CUD but also included some adult-sampled studies. This was, however, explicitly stated in the text and was solely done to describe the broad effects and potential therapeutic value of cannabis, which has not been studied in adolescents.

As for future research, we recommend that qualitative research measures (e.g., interviews) are used to explore the reasons why adolescents use cannabis and what maintaining factors of use are. Once we better understand the reasons why adolescents use cannabis, we can help them search for healthier alternatives. Also, we recommend that a future systematic review specifically researches longitudinal data on the lasting effects of adolescent cannabis use, both negative and positive.

## 5 Conclusions

Cannabis is one of the most popular drugs of the 21st century, especially among adolescents and young adults. During adolescence, the brain continues to develop, and these neurobiological changes can, in combination with social influences, lead to both adaptive goal-shifting healthy exploration and risk taking (e.g., using cannabis). Although cannabis is perceived by adolescents to be less harmful than other substances, there is evidence that it has a variety of lasting neuropsychological effects. Long-term and heavy cannabis use may result in changes in cognitive function that can subsequently jeopardize educational performance. However, the persistence of cognitive deficits after a period of abstinence and the exact relationship between cannabis use and cognitive functioning are still under debate. Furthermore, early and regular cannabis use is a predictor for increased risk of CUD and other mental health issues. Several treatment options exist for adolescents with CUD. However, a considerable proportion of adolescents with CUD do not benefit from current treatment options. Our goal was to propose a theoretical model of adolescent CUD, based on Zinberg’s drug, set, and setting model, explicated by a review of the literature on adolescent cannabis use to improve the prevention and treatment of CUD for adolescents. The literature reveals that cognitive functioning and school performance are important psychosocial determinants for adolescent cannabis use. In this paper, we propose an integrated model (Figure 2) of adolescent cannabis use in which cannabis use, executive function as part of adolescent brain development, and school performance are multidirectionally interrelated. The multidirectional relationship between adolescent cannabis use, adolescent brain development, and school performance is influenced by the properties and mechanisms of cannabis (drug), and individual (set) and environmental risk factors (setting). We argue that by paying attention to the specific combination of drug, set, and setting of an individual, better treatment outcomes are more likely to be achieved. As for preventive interventions, we propose school-based interventions during which attention must be paid to the combination of individual and environmental risk factors and more knowledge about the adolescent brain and cannabis risk among care takers and adolescents must be created. Regarding therapeutic interventions, we propose motivational enhancement therapy to help individuals resolve their possible ambivalence about engaging in CUD treatment while paying attention to the specific combination of drug, set, and setting of the individual. In addition, acceptance and commitment therapy could help target underlying mental health issues and maladaptive coping. However, further studies of this kind of tailor-made interventions are required and should also include long-term follow-up studies to investigate effectiveness over time.

## Figures and Tables

**Table 1:** DSM-5 criteria of CUD.

1.	Taking more cannabis than was intended
2.	Difficulty controlling or cutting down cannabis use
3.	Spending a lot of time obtaining, using, or recovering from cannabis
4.	Craving cannabis
5.	Problems at work, school, and home as a result of cannabis use
6.	Continuing cannabis use despite related social or relationship problems
7.	Giving up or reducing other activities in favour of cannabis use
8.	Taking cannabis in high-risk situations
9.	Continuing to use cannabis despite physical or psychological problems
10.	Tolerance to cannabis
11.	Withdrawal symptoms when discontinuing cannabis

At least 2 of these 11 factors must occur within a 12-month period. Mild: 2−3 symptoms; moderate: 4−5 symptoms; severe: >5 symptoms [23].
